# Oral inoculation of *Fusobacterium nucleatum* exacerbates ulcerative colitis via the secretion of virulence adhesin FadA

**DOI:** 10.1080/21505594.2024.2399217

**Published:** 2024-09-02

**Authors:** Donghao Li, Zongwei Li, Lei Wang, Yan Zhang, Shoubin Ning

**Affiliations:** a Department of Gastroenterology, Air Force Medical Center of Chinese People’s Liberation Army, Beijing, China; b Department of Gastroenterology, The First Medical Center of Chinese People’s Liberation Army General Hospital, Beijing, China

**Keywords:** Oral pathogens, *Fusobacterium nucleatum*, FadA adhesin, E-cadherin, ulcerative colitis

## Abstract

*Fusobacterium nucleatum* (*F. nucleatum*), an anaerobic resident of the oral cavity, is increasingly recognized as a contributing factor to ulcerative colitis (UC). The adhesive properties of *F. nucleatum* are mediated by its key virulence protein, FadA adhesin. However, further investigations are needed to understand the pathogenic mechanisms of this oral pathogen in UC. The present study aimed to explore the role of the FadA adhesin in the colonization and invasion of oral *F. nucleatum* in dextran sulphate sodium (DSS)-induced colitis mice via molecular techniques. In this study, we found that oral inoculation of *F. nucleatum* strain carrying the FadA adhesin further exacerbated DSS-induced colitis, leading to elevated alveolar bone loss, disease severity, and mortality. Additionally, CDH1 gene knockout mice treated with DSS presented increases in body weight and alveolar bone density, as well as a reduction in disease severity. Furthermore, FadA adhesin adhered to its mucosal receptor E-cadherin, leading to the phosphorylation of β-catenin and the degradation of IκBα, the activation of the NF-κB signalling pathway and the upregulation of downstream cytokines. In conclusion, this research revealed that oral inoculation with *F. nucleatum* facilitates experimental colitis via the secretion of the virulence adhesin FadA. Targeting the oral pathogen *F. nucleatum* and its virulence factor FadA may represent a promising therapeutic approach for a portion of UC patients.

## Introduction

Ulcerative colitis (UC) is a chronic and remittent intestinal inflammatory disease with a complicated and multifactorial aetiology. The colon and rectum are typical inflamed regions in UC [1]. Genetics, environment, intestinal microbiota dysbiosis, and aberrant host immunity are implicated in the pathogenesis of UC [2]. A growing body of evidence suggests that dysregulation of the gut microbiota significantly contributes to the development and pathogenesis of UC, which is characterized by a decrease in beneficial probiotics, an increase in pathogenic bacteria, and reduced microbial diversity [3–5]. As the second largest microbial ecosystem in the human body, the role of the oral microbiota in gastrointestinal diseases remains largely unknown [6,7]. Although the gut microbiota and oral microbiota possess a certain degree of similarity in the composition of microbial communities, oral and intestinal microbiota features are well segregated due to the oral-gut barrier [8]. Nevertheless, compromised epithelial barrier integrity might facilitate the translocation of oral virulent bacteria to establish colonies in the intestinal mucosa [9]. The enrichment of orally transmitted virulent bacteria in the gut therefore influences the microbial ecosystem and eventually modulates the pathogenesis of intestinal diseases [9]. Currently, a concept of the “oral-gut microbiota axis” has been proposed to elucidate the intricate and reciprocal communications between the two ecosystems [10,11].

Periodontal disease is a complex inflammatory condition triggered by dental plaque, with gingivitis serving as a precursor to its progression [12]. An imbalance of the microbiota in the gingival environment triggers host immune responses, promotes the sustained presence of pathogenic microorganisms, leads to excessive activation of immune responses and long-term inflammatory states, and promotes irreversible damage to the gums, periodontal ligament, and alveolar bone loss [13]. *Porphyromonas gingivalis* and *F. nucleatum* are the primary pathogens of periodontal disease. A higher prevalence of highly invasive and virulent strains of *Porphyromonas gingivalis* and *F. nucleatum* was observed in patients with periodontal disease [14,15]. The diseased oral cavity can serve as a reservoir for these oral pathogens [16], and the translocated pathogens may potentially act as direct exacerbating factors for intestinal inflammation [17,18].

The gram-negative opportunistic pathogen *F. nucleatum*, commonly found in the oral cavity, plays an important role in orchestrating biofilms that contribute to periodontal diseases [19]. The adhesive properties of *F. nucleatum* are prerequisites for its pathogenicity, while the attachment and invasion functions are facilitated by the adhesin FadA, a virulence factor expressed on the bacterial surface [20,21]. This adhesin is encoded by the *fadA* gene, which consists of 129 amino acid residues and an 8-amino acid signal peptide. The FadA adhesin exists in two forms: the non-secreted premature FadA and the mature FadA [22]. The spiral structure of the m-FadA monomer is hairpin-like, and the hydrophobic signal peptide acts as a hook that is cross-linked with adjacent FadA filaments to form a stable amyloid structure. As a scaffold for biofilm formation, it improves the acid resistance of bacteria and their ability to respond to external stimulation [23]. E-cadherin is a component of the tight junctions between intestinal epithelial cells and plays an important role in maintaining the integrity of the intestinal barrier. As a calcium-dependent cell adhesion glycoprotein, each E-cadherin protein is composed of five extracellular repeat domains (EC1-EC5), a transmembrane domain, and a highly conserved cytoplasmic tail [24]. It has been reported that FadA facilitates adhesion and invasion by binding to the extracellular EC5 domain of E-cadherin, leading to intracellular β-catenin phosphorylation. Phosphorylated β-catenin is subsequently translocated to the nucleus, where it regulates transcriptional responses and exerts proinflammatory or carcinogenic effects [25].

In our previous study, we established a correlation between the presence of *fadA*-carrying *F. nucleatum* in faecal samples and the severity and location of UC. These findings suggest that strains of *fadA*-positive *F. nucleatum* may participate in the pathogenesis of UC [26]. In this study, we aimed to investigate whether oral infection of *F. nucleatum* exacerbates DSS-induced colitis through its virulence adhesin FadA by establishing UC mouse models and utilizing multiple molecular techniques to analyse the underlying mechanisms involved. Additionally, we employed a transgenic mouse model to validate our hypothesis. Our findings provide insight into how the oral *F. nucleatum* virulence adhesin FadA participates in the progression of UC and shed light on the potential therapeutic strategies of UC targeting oral-associated pathogens and virulence factors in the future.

## Materials and methods

### Bacterial culture and treatment

The *F. nucleatum* strains, including the *fadA* gene-harbouring ATCC 25,586 and the *fadA*-deficient ATCC 12,230-US1, were utilized in this study. Both ATCC 25,586 and ATCC 12,230-US1 were inoculated on blood agar medium after 2 days of anaerobic culture at 37°C. A single colony was then transferred to 10 ml BHI medium containing vitamin K (0.2 μg/ml) and haem chloride (5 μg/ml) for a period of 2 days. Following centrifugation at 4000 r/min for 10 minutes, the collected cells were washed with PBS, subjected to another round of centrifugation for 10 minutes, and subsequently diluted with PBS to achieve a concentration of 1 × 10^9^ CFU/ml.

### Construction of a colitis animal model

This study was approved by the Ethics Committee of the Air Force Medical Center of the Chinese People’s Liberation Army (Approval ID 2023–246-S01). Animal experiments were conducted in compliance with ARRIVE guidelines (ARRIVE 2.0) and international laws and policies (Guide for the Care and Use of Laboratory Animals). The experimental animals used for the study were specific-pathogen-free (SPF) male C57BL/6 mice (6–8 weeks, 20–22 g). CDH1 gene knockout (CDH1^KO^) mice were obtained from Cyagen Biosciences. The mice were raised in a standard laboratory environment and had free access to food and water. To ensure microbiota consistency and promote the colonization of *F. nucleatum*, each group of mice was given drinking water supplemented with 2 mg/ml streptomycin for three days before the colitis model was induced. In this study, DSS was dissolved in drinking water to prepare a 2.5% (w/v) solution for C57BL/6 mice to drink for 14 days. Thirty-two C57BL/6 mice were randomly assigned to four groups (*n* = 8 in each group), Healthy control (HC), DSS group (DSS), oral inoculation ATCC 12,230-US1 group (OR-1), and oral inoculation ATCC 25,586 group (OR-2). Eight transgenic mice (CDH1^KO^) were also included in the study. The mice in the HC group had free access to normal food and water. The mice in the DSS, OR-1, OR-2, and CDH1^KO^ groups were orally administered a 2.5% DSS solution consecutively for a duration of 14 days. In addition, mice in OR-1 were brushed daily with ATCC 12,230-US1 solution in the oral cavity. Mice in the OR-2 and CDH1^KO^ groups were daily brushed with the ATCC 25,586 solution in the oral cavity. The mice were orally inoculated with an *F. nucleatum* suspension at a normal dose of 10 ml/kg, which was adjusted according to changes of body weight. The inoculation process involved the use of a small brush to apply *F. nucleatum* solution, ensuring full coverage of the oral cavity, particularly the gingival surface. This was done by gently and carefully brushing twice. Furthermore, the mice were fasted for 2 hours after each inoculation to prolong the residence time of *F. nucleatum* in the oral cavity.

**Figure 1.**
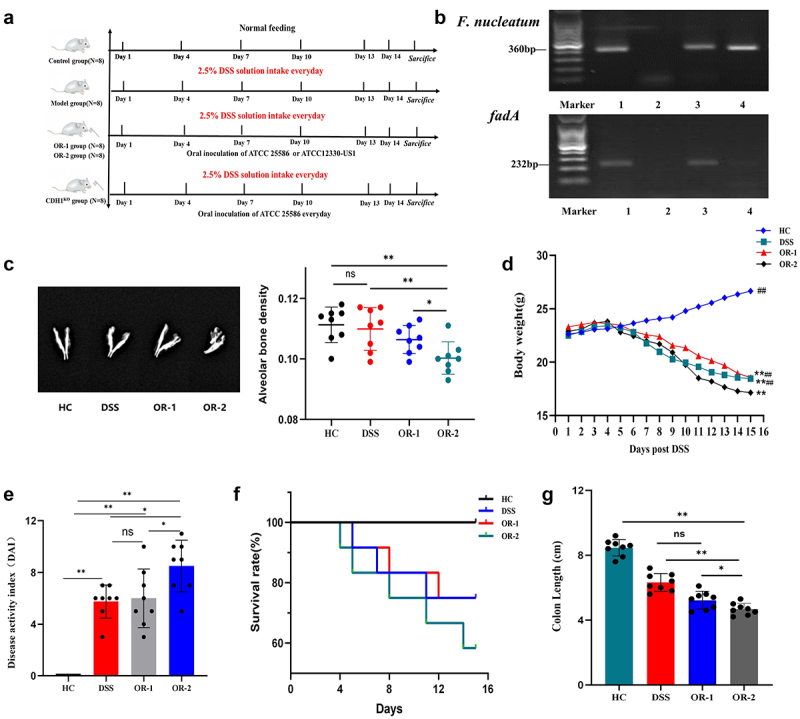
*F. nucleatum* strains that harbour the virulence gene *fadA* are more likely to exacerbate UC in mice than the *fadA*-deficient strain.

The mice were euthanized 24 hours following the final oral inoculation. Daily body weight, disease activity index (DAI) and survival rates were recorded. Colon length was measured and promptly divided into multiple segments, which were fixed in a 10% formalin solution. The remaining segments were stored in a −80°C refrigerator for subsequent Western blotting and RT-PCR analysis. Maxillaries were collected and preserved in PBS for alveolar bone density assessment. A flowchart illustrating the animal experiments conducted in this study is presented in Figure 1a.

(a) A flowchart of the animal experiments. (b) Results of *F. nucleatum* by 16S rRNA PCR (shown in the top figure) and the *fadA* gene by PCR (shown in the bottom figure). Lane 1: positive control for *F. nucleatum*; Lane 2: negative control; Lane 3: sample of ATCC 25,586; Lane 4: sample of ATCC 12,230-US1. Bottom figure, Lane 1: Positive control of *fadA*; Lane 2: Negative control; Lane 3: Sample of ATCC 25,586; Lane 4: Sample of ATCC 12,230-US1. (c) Dual-energy X-ray absorptiometry showing the density of alveolar bone among the four groups. (d) Time-dependent changes in body weight in the four groups of mice (**p < 0.01 vs HC, ^##^*p* < 0.01 vs OR-2). (e) Comparison of the DAI in different groups of mice after 14 days of intervention revealed that the OR-2 group presented more severe disease activity than the OR-1 and DSS groups did. (f) Survival rates of the four groups after 14 days of treatment. (g) Comparison of colon length among the different groups of mice. ns: not significant, *p < 0.05, **p < 0.01.

### Measurement of the severity of colitis

The mice were observed daily for body weight, mental status, mortality, stool consistency, and the presence of perianal blood. Disease activity was evaluated via an established scoring system [27]. An unbiased observer was used to assess the disease activity index (DAI) score via the scoring system. The DAI was calculated as follows: (a) weight loss degree: 0 (no loss), 1 (1–5%), 2 (5–10%), 3 (10–20%), and 4 (>20%); (b) stool formability: 0 (normal), 2 (sparse stool), 4 (diarrhea); (c) degree of hematochezia: 0 (no hematochezia), 1 (positive for occult blood test), 2 (positive for occult blood test and a small part of bloody stool can be seen with the naked eye), 4 (obvious bloody stool and bleeding around the anus). DAI= weight loss+stool formability+hematochezia.

### Alveolar bone density measurement

After the mice were euthanized, the alveolar bones were isolated and immersed in a PBS solution for subsequent analysis. Using dual-energy X-ray (DEXA) imaging software, the alveolar bone of each mouse was subjected to scanning, followed by delineation of regions exhibiting similar morphology and structure for further image analysis. Finally, the bone mineral density of the alveolar bone was quantified.

### Histopathological assessment

The distal colon specimens were collected and fixed in 10% buffered formalin for a duration of 24 hours. H&E staining was subsequently conducted to visualize the tissue samples. The histopathological score was determined by assessing the extent of mucosal damage, loss of glandular mucosa, tissue injury, and infiltration of inflammatory cells.

### Immunohistochemical staining

E-cadherin and occludin proteins in colon specimens were stained via immunohistochemistry. In brief, colon tissues that were first embedded in conventional paraffin were incubated with the primary antibodies anti-E-cadherin (Abcam) and anti-occludin (Abcam) in a wet box at 4^◦^C overnight. Horseradish peroxidase-labelled secondary antibodies were incubated for 50 minutes at room temperature. Antigen-antibody binding was detected using 3,3’-diaminobenzidine (DAB). DAB staining was analysed via Image Pro Plus software (Media Cybernetics).

### Immunofluorescence staining

The colon tissues were dehydrated using gradient alcohol, embedded in paraffin, and then sliced. Antigen retrieval was performed in an electric ceramic furnace. Normal goat serum was added for antigen retrieval and incubated at room temperature for 30 minutes. Following the addition of the primary antibody (FadA rabbit antibody, 1:200) from Abcam, incubation took place in a humidified chamber at 4°C overnight (15 hours). A diluted fluorescent secondary antibody (CY3 labelled sheep anti-Rabbit IgG, 1:100) was applied and incubated in a humidified chamber at 37°C for 1 hour. The sections were washed with PBST four times, followed by incubation with DAPI for 5 minutes in the dark to stain the nuclei. The slides were subsequently sealed using a mounting solution containing an anti-fluorescence quencher before the acquired images were observed under a fluorescence microscope.

### RT-PCR

The colon tissue samples were cryogenically ground in a mortar with liquid nitrogen until thoroughly homogenized. Total DNA and RNA extraction from the colons was performed using the TIANamp DNA Kit (TIANGEN) and Qiagen RNeasy Mini Kit (Qiagen), respectively, following the manufacturer’s protocol. Subsequently, the extracted RNA was reverse transcribed into cDNA for RT-PCR analysis. Genomic DNA from *F. nucleatum* was isolated using the TIANamp Bacteria DNA Kit. Prior to RT-PCR, the concentration, purity, and integrity of both extracted DNA and cDNA were assessed. The RT-PCR reaction system consisted of SYBR Green I Master Mix (TAKARA), upstream and downstream primers, templates of either DNA or cDNA, ultrapure water, and Step-One Plus™ instrument (Thermo Fisher). The reaction conditions were as follows: 95°C for 10 min; 95°C for 15 s, 60°C for 15 s, 72°C for 30 s, and 72°C for collecting fluorescence for a total of 40 cycles. The primer set for *F. nucleatum* was designed to target the conserved *nusG* gene. The primer sequences targeting the *nusG* and *fadA* genes used in this experiment were designed with Primer Premier software (PREMIER) according to the primer design guidelines, and synthesized by Sangon Biotech. *nusG* Forward: 5′-TGG AAA CAA CTC AGC AGC GAA GG-3,′ Reverse: AGG CGT TGG TTG ATC TGT GTC AC-3;′ *fadA* Forward: 5′-CTA TTG TGC TGC CCT TCT CCT TCC-3,′ Reverse: 5′-GCT GCT GCT CAT CGC CGA TAT AG-3;′ the primer sequences for mRNA were as described in the literature [28,29]. E-cadherin(mRNA) Forward: 5′-CAT AAA CGA GGT TCT GTC TTC A-3,′ Reverse: 5′-GGA TAA TAG TCG GGA GGT GTT-3;′ β-catenin(mRNA) Forward: 5′-GAC CAC AAG CAG AGT GCT GA-3,′ Reverse: 5′-ACT CGG GTC TGT CAG GTG AG-3;′ IκBα (mRNA) Forward: 5′-GCA AAA TCC TGA CCT GGT GT-3,′ Reverse: 5′-GCT GGT CCT CTG TGA ACT CC-3;′ NF-κB(mRNA) Forward: 5′-CGG AAT GTG CAG ATG-3,′ Reverse: 5′-ACC CCC ACT ACT CTT GCG GCA-3.′

### Western blot

Colon tissues were subjected to ice-cold homogenization via proteinase digestion solution. After 30 minutes of homogenization and 30 minutes of centrifugation at 12,000/rpm, the supernatant was collected. The Bicinchoninic acid (BCA) method was used to measure the protein concentration. A total of 40 μg of protein was added to the sample solution and subjected to denaturation at 100°C for 5 minutes. The sample was then electrophoresed on a 10% SDS-PAGE gel at 90 mA and 250 mA. The gel was electro-transferred to a PVDF membrane using a semi-dry method with 5% milk dropout. After 1 hour at room temperature blocking with 5% skim milk, the membrane was incubated with the corresponding primary antibodies (Abcam, E-cadherin 1:1000, β-catenin 1:1000, and GAPDH 1:2000). The membrane was washed three times with TBST for 5 minutes, followed by incubation with HRP-labelled secondary antibody (goat anti-rabbit 1:4000) for 1 hour at room temperature. The membrane was washed 5 minutes with TBST for three times, and enhanced chemiluminescence (ECL) chemical development was used to visualize the developed image. Image-Pro Plus software was used to perform cumulative absorbance at 450 nm (IA) analysis of the developed image, and the relative expression level of the target protein was calculated as the ratio of the target protein to the internal reference protein GAPDH.

### Enzyme-linked immunosorbent assay

The levels of NF-κB inflammatory cytokines in the colon were assessed using an enzyme-linked immunosorbent assay (ELISA) kit (Solarbio). Initially, the colon samples were weighed and ground in a bowl containing liquid nitrogen to extract colonic protein. Subsequently, ultrasonic crushing was employed to disrupt the structure of the colon with PBS solution. Following centrifugation, the supernatant was collected for subsequent experiments. In accordance with the instructions provided with the kit, TNF-α, IL-1β, IL-6, and IL-8 levels in mouse colons were determined using a Microplate Spectrophotometer (BioTek).

### Statistical analysis

Pearson’s chi-square test and Fisher’s exact test were used to analyse categorical variables. One-way ANOVA was used to compare groups conforming to the normal distribution and homogeneity of variance. The measurement data that were not normally distributed were analysed via nonparametric tests (Kruskal‒Wallis tests). Two-variable correlation analysis was performed via Spearman rank correlation analysis. All the statistical analyses were conducted via IBM SPSS Statistics 22.0. A value of *p* < 0.05 was considered statistically significant.

## Results

### Oral inoculation of ATCC 25,586 but not ATCC 12,230-US1 exacerbated colitis in DSS mice

To investigate the role of FadA adhesin in contributing to the progression of UC, we orally inoculated *fadA*-positive (ATCC 25,586) or *fadA*-negative (ATCC 12,230-US1) strains to DSS-treated mice. Our results demonstrated that the oral infection of ATCC 25,586 is more likely to exacerbate colitis compared to ATCC 12,230-US1. The two strains were initially identified using PCR. The results revealed that ATCC 25,586 exhibited the presence of the *fadA* gene, while ATCC 12,230-US1 did not demonstrate its expression (Figure 1b). Mice alveolar bone mineral density was measured. The alveolar bone mineral density in OR-2 mice was significantly lower compared to that in HC, DSS mice (*p* < 0.01), and OR-1 mice (*p* < 0.05). However, there was no significant difference observed between the DSS and OR-1 mice (Figure 1c). In the HC group, there was a progressive increase in body weight over time, while mice in the DSS, OR-1, and OR-2 groups exhibited significant progressive decreases in body weight, with the most pronounced decrease observed in the OR-2 group (*p* < 0.01, Figure 1d). The disease activity index (DAI) was also calculated. The OR-2 mice exhibited severe diarrhoea, hematochezia, perianal bleeding, and higher DAI scores (*p* < 0.01). There was no significant difference in DAI scores between the DSS and OR-1 mice groups (Figure 1e). Regarding survival rate, none of the mice in the HC group succumbed to the experimental conditions. The mortality rate of OR-2 mice was significantly higher compared to that of the DSS and OR-1 groups (*p* < 0.01, Figure 1f). Furthermore, colon length in OR-2 mice was shorter than that observed in HC, DSS, and OR-1 mice (*p* < 0.05, Figure 1g).

### Oral inoculation of ATCC 25,586 but not ATCC12230-US1 facilitated the damage to the intestinal barrier

**Figure 2.**
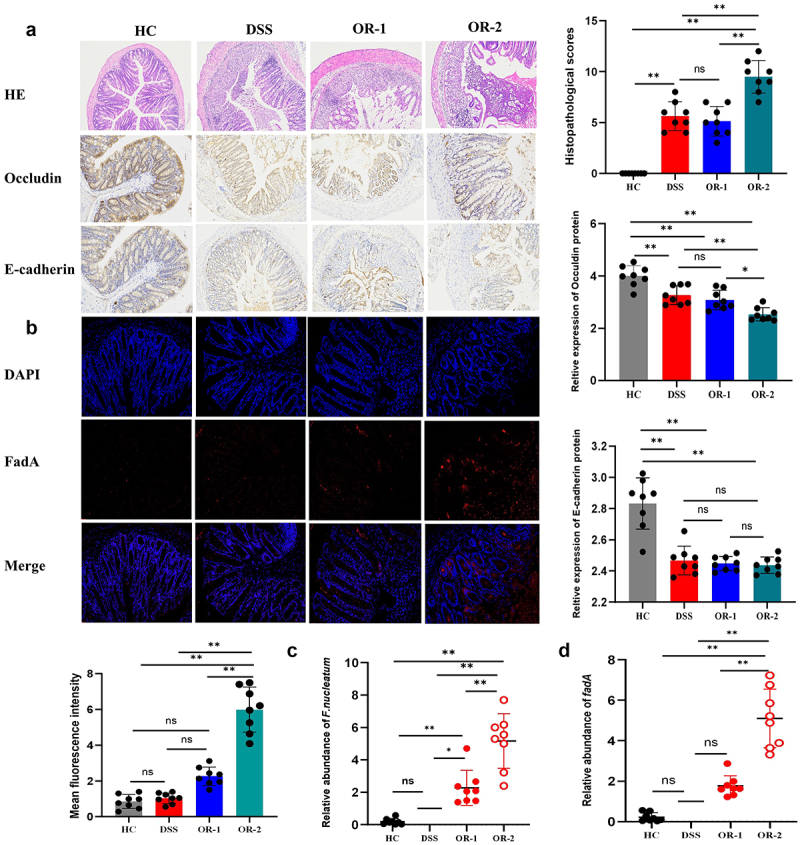
Strain-harboured *fadA* exacerbates histopathological lesions and damages the intestinal mucosal barrier in the UC mice model, in contrast to the *fadA*-deficient strain, while also promoting the colonization of *F. nucleatum* in the colon.

We used histopathological staining and immunohistochemical methods to assess the impact of different strains of infection on intestinal barrier damage. The histopathological examination of the colon demonstrated that the intestinal epithelium and structure of HC mice remained intact, with no evidence of inflammatory cell infiltration. Compromised integrity of the intestinal epithelium and scattered infiltration by inflammatory cells were observed in both DSS and OR-1 mice. The intestinal integrity and structural damage were more severe in OR-2 mice, as evidenced by the presence of mucosal gland deletion and a significant infiltration of inflammatory cells in both the mucosal and submucosal layers (Figure 2a). Histopathological scores were consistent with the histopathological results. To investigate the function of the intestinal mucosal barrier, immunohistochemical staining was utilized to detect occludin, a tight junction protein. In HC mice, occludin protein exhibited intact and continuous expression in the intestinal epithelium; however, its expression showed noticeable disruption and decreased levels in DSS and OR-1 mice. Moreover, the expression in OR-2 mice exhibited a significantly lower level compared to that observed in DSS and OR-1 mice (*p* < 0.05, Figure 2a). The immunohistochemical staining of E-cadherin revealed that the expression was integral and sustained in the HC group, whereas it showed a significant decrease in DSS, OR-1, and OR-2 mice (*p* < 0.01). However, no significant differences were observed among the three groups (Figure 2a). Notably, the colons of OR-2 mice exhibited a distinct distribution pattern of FadA protein with a significantly higher mean fluorescence intensity compared to HC, DSS, and OR-1 mice (*p* < 0.01). In contrast, OR-1 mice displayed scattered fluorescence signals similar to those observed in the HC and DSS groups (*p* > 0.05, Figure 2b). The RT-PCR results revealed a significant upregulation of the relative gene level of *nusG* in OR-2 mice compared to HC, DSS, and OR-1 mice (*p* < 0.01). Conversely, OR-1 mice exhibited higher relative expression levels than the HC and DSS groups (*p* < 0.05, Figure 2c). The level of *fadA* in OR-2 mice was significantly higher than that in HC, DSS, and OR-1 mice (*p* < 0.01), while no difference was observed between HC and DSS mice in OR-1 group (*p* > 0.05, Figure 2d).

(a) Representative H&E staining images and comparison of histopathological scores and immunohistochemical staining results for occludin and E-cadherin proteins in each group. (b) Immunofluorescence staining results showing the distribution of mucosal FadA adhesin. (c, d) Relative expression levels of the colonic *nusG* and *fadA* genes in each group.

### CDH1 gene knockout attenuated the facilitating effects of *F. nucleatum* on disease activity and gut barrier damage

**Figure 3.**
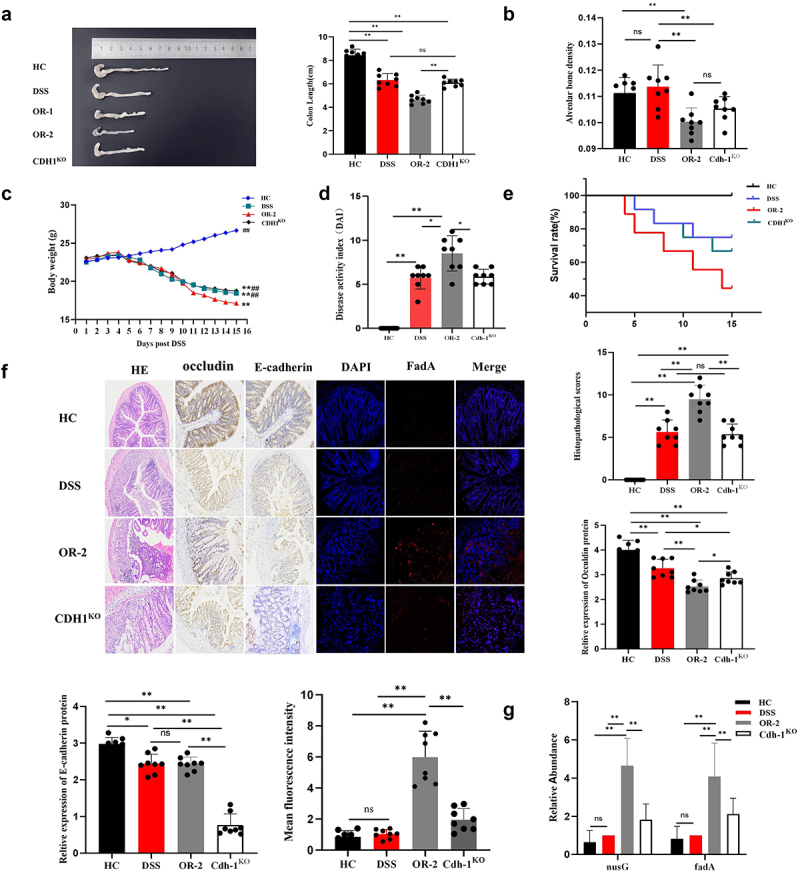
The promoting effect of *F. nucleatum* on disease severity and intestinal mucosal barrier damage was weakened after CDH1 gene knockout.

To investigate whether the E-cadherin protein is involved in the pro-inflammatory process of *F. nucleatum*, we knocked out the CDH1 gene in C57BL/6 background mice to construct an E-cadherin-deficient mouse model. By orally inoculating C57BL/6 mice and transgenic mice with ATCC 25,586, we investigated the impact of *F. nucleatum* on DSS-induced colitis. The results demonstrated that the colon length of CDH1^KO^ mice was greater than that of OR-2 mice, yet shorter compared to HC mice (*p* < 0.01, Figure 3a). No significant difference in colon length was observed between CDH1^KO^ and DSS mice (*p* > 0.05, Figure 3a). The alveolar bone density in OR-2 mice was significantly lower compared to HC and DSS mice, but there was no statistically significant difference when compared to CDH1^KO^ mice (*p* > 0.05, Figure 3b). The body weight of CDH1^KO^ mice gradually decreased over time, although the decrease was not as pronounced as that observed in OR-2 mice (*p* < 0.01, Figure 3c). Regarding disease severity assessed by DAI, OR-2 mice exhibited the most severe symptoms (*p* < 0.05), while no significant difference was found between CDH1^KO^ and DSS mice (*p* > 0.05, Figure 3d). The OR-2 mice exhibited the highest mortality rate and a significantly pronounced declining trend in the survival curve, whereas CDH1^KO^ mice displayed a downward trend compared to OR-2 mice (*p* < 0.01, Figure 3e). H&E staining revealed that CDH1^KO^ mice maintained continuous intestinal epithelium and intact crypt structure with scattered infiltration of inflammatory cells, while OR-2 mice suffered severe damage characterized by extensive mucosal injury, loss of crypts, and widespread infiltration of inflammatory cells. Histopathological scores showed no significant difference between CDH1^KO^ and DSS mice but were lower than those observed in OR-2 mice (*p* < 0.01, Figure 3f). Immunohistochemical staining for occludin demonstrated varying degrees of damage and disrupted expression in DSS, OR-2, and CDH1^KO^ mice; however, the disruption was most prominent in OR-2 mice. In addition, occludin expression in CDH1^KO^ mice was slightly lower than that in DSS mice and significantly lower than that in HC mice (*p* < 0.01, Figure 3f). Immunohistochemical staining results demonstrated that E-cadherin expression in the colon of CDH1^KO^ mice was significantly reduced compared to other three groups (*p* < 0.01). DSS and OR-2 mice were lower than HC mice; however, there was no significant difference between the two groups (*p* > 0.05). In addition, OR-2 mice exhibited a pronounced distribution of the FadA protein in the colon, with significantly higher mean fluorescence intensity compared to HC, DSS, and CDH1^KO^ mice (*p* < 0.01, Figure 3f). No significant differences were observed among HC, DSS, and CDH1^KO^ mice (*p* > 0.05, Figure 3f). RT-PCR results revealed a significantly elevated relative level of the *nusG* gene in OR-2 mice compared to HC, DSS, and OR-1 mice (*p* < 0.01, Figure 3g). Furthermore, the expression level of the *fadA* gene was higher in OR-2 mice than in HC, DSS and CDH1^KO^ mice (*p* < 0.01, Figure 3g).

(a) Comparison of colon length among the different groups. (b) Mouse alveolar bone density measured by dual-energy X-ray absorptiometry. (c) Body weight changes after 14 days of treatment (**p < 0.01 vs HC, ^#^*p* < 0.05, ^##^*p* < 0.01 vs OR-2). (d) Comparison of DAI among the four groups. (e) Survival rates of the four groups after 14 days of treatment. (f) Comparison of histopathological scores and the expression of occludin, E-cadherin, and FadA adhesin among the four groups. (g) The relative gene expression levels of colonic *nusG* and *fadA* in each group. ns: not significant, *p < 0.05, **p < 0.01.

### The FadA/E-cadherin/β-catenin interactions regulated the NF-κB signaling pathway to aggravate colitis

**Figure 4.**
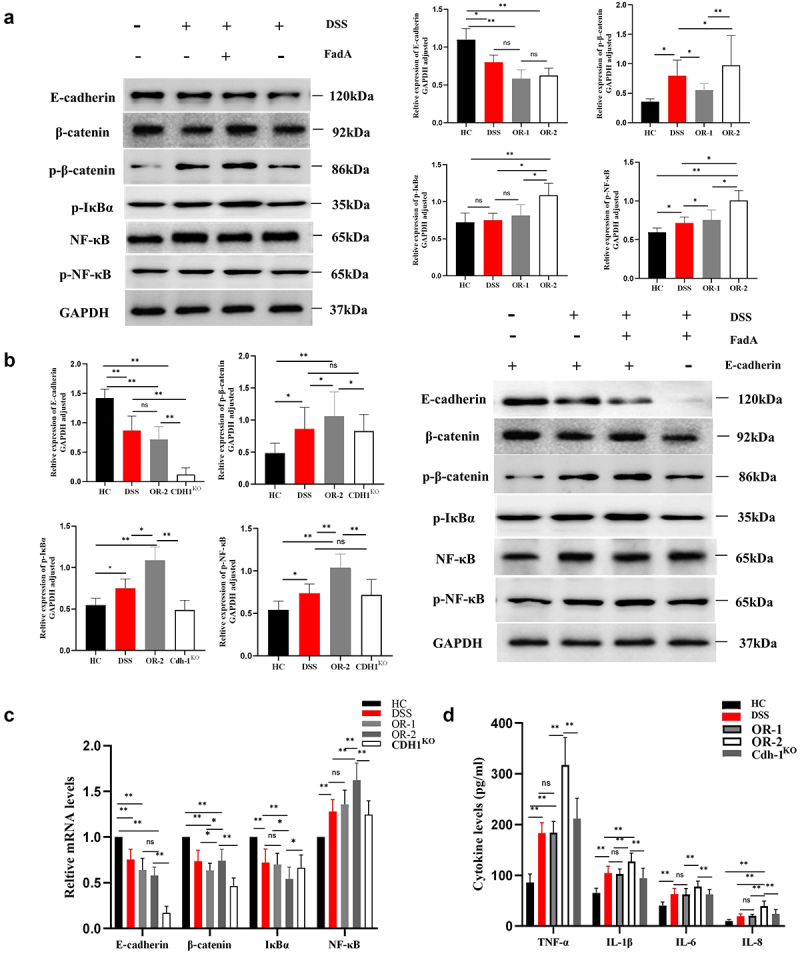
FadA adhesin regulates E-cadherin on the cell membrane and phosphorylates β-catenin and IκBα proteins to activate the nf-κB signalling pathway to aggravate colitis.

To investigate the underlying mechanisms of FadA adhesin in disease pathogenesis, we carried out relevant genetic and proteinic level investigations. Western blot analysis revealed a downregulation of colon E-cadherin protein levels in DSS, OR-1, and OR-2 mice; however, no significant differences were observed among these three groups (*p* > 0.05, Figure 4a). Similarly, there were no significant variations in β-catenin levels across all groups (*p* > 0.05, Figure 4a). Nevertheless, phosphorylated β-catenin levels exhibited a significant increase specifically in OR-2 mice compared to the other three groups (*p* < 0.05, Figure 4a). Furthermore, phosphorylated β-catenin was also elevated in DSS and OR-1 mice when compared to HC mice (*p* < 0.05, Figure 4a). The strongest phosphorylation of IκBα protein was observed in OR-2 mice as demonstrated by increased p-IκBα levels – an important indicator for NF-κB activation. Additionally, p-NF-κB levels were significantly elevated in DSS mice, OR-1, and OR-2 mice, and phosphorylation levels were highest in OR-2 mice (*p* < 0.05, Figure 4a).

(a, b) The protein levels of E-cadherin, β-catenin, p-IκBα, and NF-κB in colon samples were determined via Western blotting. (c) mRNA levels of E-cadherin, β-catenin, IκBα, and NF-κB in each group of mice. (d) Cytokine levels of TNF-α, IL-1β, IL-6, and IL-8 in colon tissues were measured via ELISA. ns: not significant, *p < 0.05, **p < 0.01.

We also observed significantly attenuated the expression of E-cadherin in transgenic mice. Besides, the downregulation of phosphorylated β-catenin was observed in transgenic mice (Figure 4b). Notably, transgenic mice exhibited reduced levels of p-IκBα protein and p-NF-κB compared to OR-2 mice (*p* < 0.01, Figure 4b). Correspondingly, there was a significant decrease observed in the levels of E-cadherin mRNA in the transgenic mice. Both OR-1 and OR-2 mice exhibited a statistically significant reduction compared to HC mice (*p* < 0.05, Figure 4c). The transgenic mice demonstrated a significant reduction in β-catenin mRNA levels compared to the other groups, whereas an increased level was observed in OR-2 mice relative to DSS and OR-1 mice (*p* < 0.05, Figure 4c). The expression levels of IκBα mRNA were the lowest in OR-2 mice, while no significant differences were observed among DSS, OR-1, and CDH1^KO^ mice. Additionally, OR-2 mice exhibited the highest levels of NF-κB mRNA, whereas DSS, OR-1 and CDH1^KO^ mice displayed significantly higher levels compared to HC mice (*p* < 0.05, Figure 4c). ELISA analysis revealed a significant increase in cytokines (TNF-α, IL-1β, IL-6, and IL-8) in OR-2 mice (*p* < 0.01, Figure 4d). Besides, TNF-α levels were higher in CDH1^KO^ mice than those observed in DSS and OR-l mice.

## Discussion

The oral and intestinal microbiota are the two largest microbial ecosystems in the human body. In recent years, the concept of the “oral-gut microbiome axis” has drawn extensive attention [10]. The presence of barriers such as gastric acid, bile acid, and rapid peristalsis in the small intestine hinders direct connections between the two microbial populations. However, it is reasonable to speculate that associations exist between oral and gastrointestinal microbiota due to their anatomical connections. Recent findings suggest that *F. nucleatum* is likely to be an important mediator connecting oral microbiota with gastrointestinal diseases, as several studies have reported the role of *F. nucleatum* in UC [17,30,31]. Lin et al. demonstrated that *F. nucleatum* exacerbates inflammation and impairs barrier function induced by DSS in experimental mice model, leading to reduced levels of symbiotic bacteria such as *bifidobacteria* and increased abundances of pathogenic bacteria such as *Escherichia coli* and *Shigella* [17]. Liu et al. reported that *F. nucleatum* can significantly exacerbate mucosal epithelial barrier damage and aggravate oxidative stress in mice. The mechanism is related to the activation of receptor-interacting protein kinase-1 (RIPK-1), the activation of RIPK1, which then mediates epithelial cell death and promotes Fn-EVs (*F. nulceatum*-secreted extracellular vesicles) to induce gut barrier disruption in UC [30]. Moreover, Su et al. demonstrated that *F. nucleatum* expresses endotoxins to promote the development of UC by inducing autophagic apoptosis in intestinal epithelial cells, indicating that *F. nucleatum* is a risk factor for high disease activity in UC patients [31]. As *F. nucleatum* is an opportunistic pathogen in the oral cavity and rarely colonizes the intestines of healthy individuals, we hypothesize that *F. nucleatum* may translocated to the intestine from the oral cavity. In this study, we administered an oral inoculation of *F. nucleatum* to investigate its impact on DSS-induced colitis. Compared with DSS mice, orally infected with ATCC 25,586 mice presented greater body weight loss, increased mortality, greater disease activity index (DAI), and more severe disruption of the epithelial barrier. Additionally, orally inoculated mice showed a higher abundance of *nusG* and *fadA* genes in the colon. These findings suggest that oral inoculation of *F. nucleatum* can result in intestinal colonization and exacerbation the severity of colitis.

As an adhesive microorganism, *F. nucleatum* can adhere to the surfaces of epithelial cells, polymorphonuclear leukocytes, and fibroblasts. FadA adhesin is the key virulence protein of *F. nucleatum* and exerts functions in the processes of adhesion, colonization, and invasion of host cells. FadA has previously been reported to promote colorectal cancer cell metastasis [25]. In this study, we conducted oral inoculation of *fadA*-positive or *fadA*-negative strains in DSS-induced colitis mice to investigate the role of FadA adhesin in *F. nucleatum* colonization and invasion. Our findings revealed that mice treated with ATCC 25,586 exhibited greater body weight loss, increased mortality, and more severe disease activity compared to those treated with ATCC 12,230-US1. Similarly, ATCC 25,586 was more likely to compromise the integrity of the intestinal mucosal barrier, as evidenced by histopathological analysis and reduced expression of the occludin protein. These findings confirmed the essential role of FadA adhesin in facilitating colonization and pathogenicity of *F. nucleatum* within the intestinal mucosa.

The adhesin FadA, however, necessitates specific receptor binding to the intestinal epithelium to fulfill its attachment function. E-cadherin is a cell surface glycoprotein that plays an important role in cell-cell adhesion. As previously reported, E-cadherin is a mucosal receptor for the FadA protein in epithelial cells [32]. The extracellular domains of E-cadherin consist of five repeat domains (EC1-EC5), with EC5 being the binding site for the FadA protein, and the cytoplasmic portion can bind to the cytoplasmic component β-catenin [32]. β-catenin combines with E-cadherin and α-catenin on the cell membrane to form a complex and mediates extracellular adhesion molecules to maintain the integrity of the cell membrane and tissue structure [32]. β-catenin also participates in the transduction of several signalling pathways such as Wnt/β-catenin and SIAH-1-SIAH interacting protein-SCFEbi-β-catenin [33]. Accumulating evidence has consistently demonstrated the involvement of β-catenin in the regulation of the NF-κB signalling pathway [34–37]. Robinson et al. revealed a significant reduction, up to 70%, in NF-κB activity due to the absence of β-catenin in human astrocytes [34]. Zhou et al. discovered that FAS ligand expression was suppressed in ischaemic kidneys following β-catenin knockout in fibroblasts, suggesting that β-catenin may exert a protective effect on the kidney and inhibit apoptosis through the NF-κB pathway [35]. The overexpression of β-catenin and its associated Wnt family proteins can initiate the activation of the Wnt/β-catenin pathway, thereby promoting IκBα degradation and subsequently activating the NF-κB signalling pathway [36]. The study conducted by Lee et al. has revealed that β-catenin potentially regulates the transcriptional activity of NF-κB. They have observed that BFT-induced β-catenin signalling occurs upstream of NF-κB activation [37]. Our findings have revealed that transgenic mice exhibited reduced mortality and disease severity, improved histopathological features, and elevated occludin expression. Furthermore, we observed increased mRNA levels of IκBα and decreased levels of β-catenin and NF-κB, along with the expression of inflammatory cytokines TNF-α, IL-1β, IL-6, and IL-8. These results demonstrated the regulatory role of FadA adhesin in intracellular β-catenin protein via E-cadherin protein, subsequently activating the NF-κB signalling pathway and upregulating inflammatory cytokines to promote UC.

Despite some meaningful findings, this study is subject to certain limitations. The two *F. nucleatum* bacterial strains utilized in the experiment do not genuinely originate from the oral cavity; ATCC 25,586 strain was isolated from a cervicofacial lesion, and ATCC 12,230 is a transtracheal isolate. Although ATCC 25,586 and ATCC 12,230 originate from cervicofacial or transtracheal lesions, these strains were confirmed to exert certain roles in the process of oral inflammation such as biofilm formation and have been employed in oral research [38,39]. Additionally, we used the ATCC 12,230-US1 strain to serve as a *fadA* defective control in the experiment. ATCC 25,586 belongs to the *F. nucleatum* subspecies *nucleatum*, whereas ATCC 12,230 is classified under the *F. nucleatum* subspecies *polymorphum*, indicating genotypic and phenotypic differences between them. We acknowledge the heterogeneity between the two strains, but we also recognize that ATCC 12,230-US1 and ATCC 25,586 strains both belong to the genus *F. nucleatum*, and they share certain bacterial characteristics and pathogenicity. To confirm this, we verified the specificity of bacteria and *fadA* gene via PCR (Figure 1b). We also acknowledge that a single pathogenic bacterium *F. nucleatum* cannot fully reflect the roles of oral virulent bacteria in gastrointestinal diseases. Further investigations incorporating other oral virulent bacteria and associated virulent factors are needed to validate these findings.

## Conclusions

**Figure 5.**
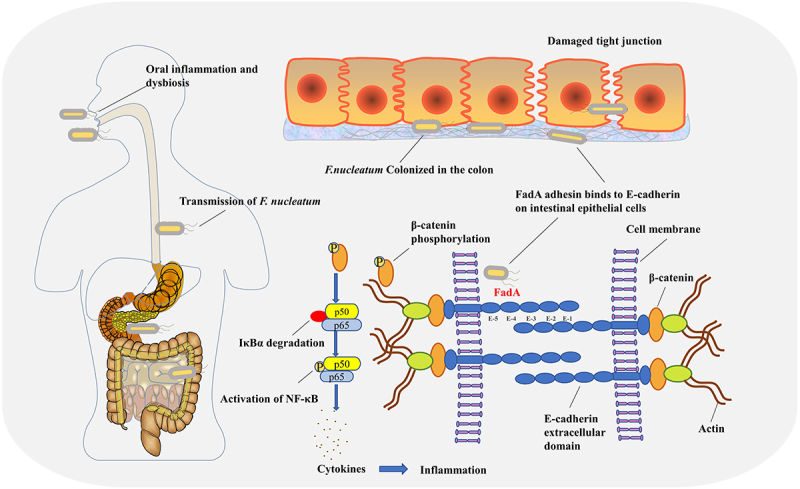
A proposed model showing how oral *F. nucleatum* translocates to the colon and promotes colitis by regulating the nf-κB signalling pathway.

In conclusion, perturbations in the gut microbiota and impairment of the mucosal barrier in UC patients diminish intestinal colonization resistance, thereby enabling oral *F. nucleatum* to colonize the intestine. By interacting with the extracellular domain E-5 of E-cadherin, the virulence FadA adhesin regulates intracellular β-catenin phosphorylation and leads to NF-κB inhibiting protein IκBα degradation, subsequently triggers the activation of the NF-κB signalling pathway and upregulation of downstream inflammatory factors, induces the exacerbation of colitis (Figure 5). Our findings elucidate the mechanisms by which *F. nucleatum* facilitates colitis through the secretion of virulence adhesin FadA. A therapeutic strategy targeting oral and intestinal *F. nucleatum* as well as the virulence adhesin FadA may offer potential benefits for a subset of individuals with UC.

Alterations in the gut microbiota and destruction of the intestinal mucosal barrier in UC patients suggest that *F. nucleatum* may be translocated to the intestine. The virulence protein FadA binds to the E-cadherin extracellular E5 domain, followed by E-cadherin phosphorylation and internalization on the cell membrane, and this process is accompanied by β-catenin phosphorylation. Phosphorylated β-catenin is then transferred to the cytoplasm, where it regulates transcriptional responses and phosphorylation of IκBα, activates the NF-κB signalling pathway, upregulates proinflammatory cytokines and exacerbates colitis in UC.

## Supplementary Material

ARRIVE_2.0_Author_Checklist-2.pdf

## Data Availability

The raw data that support the findings of this study are available in the Science Data Bank at https://doi.org/10.57760/sciencedb.15998.
